# Sulfonamide-induced acute eosinophilic pneumonia requiring extracorporeal membrane oxygenation support: a case report

**DOI:** 10.5935/2965-2774.20230404-en

**Published:** 2023

**Authors:** Ana Flávia Garcia Silva, Lívia Maria Garcia Melro, Bruno Adler Maccagnan Pinheiro Besen, Pedro Vitale Mendes, Marcelo Park

**Affiliations:** 1 Intensive Care Unit, Emergence Department, Hospital das Clínicas, Faculdade de Medicina, Universidade de São Paulo - São Paulo (SP), Brazil

## INTRODUCTION

Acute eosinophilic pneumonia (AEP) is a rare cause of acute respiratory failure that affects people aged 20-40 years old.^(1)^ Patients with AEP present with rapid onset of cough, dyspnea, tachypnea and fever of usually less than 7 days of duration. Hypoxemia is present in all cases, and most patients do not have peripheral blood eosinophilia. In contrast, an increase in eosinophils in bronchoalveolar lavage fluid (BALF) is a marker of the disease, exceeding 20% of the BALF cell count in most patients. Radiographs show mixed reticular and alveolar infiltrates, which then can progress to be densely alveolar as the condition worsens.^(2,3)^ Acute and organizing diffuse alveolar damage is common and is usually responsive to corticosteroids.^(1)^

The major causes of pulmonary eosinophilia include inhalation of antigens, such as demolition dust, cigarette smoke, electronic cigarettes, cannabis, crack cocaine; parasitic and fungal infections; HIV infection; previous irradiation of the chest; and recent use of drugs associated with pulmonary eosinophilia, such as ranitidine, venlafaxine, infliximab, phenytoin, nitrofurantoin, beta-lactam antibiotics, sulfazalazine-mesalazine, among others. Differential diagnosis includes acute interstitial pneumonia, cryptogenic organizing pneumonia, diffuse alveolar hemorrhage and granulomatosis with polyangiitis. These conditions have similar clinical presentations but without pulmonary eosinophilia.

Sulfonamide-induced AEP is described as the cause of severe acute respiratory distress syndrome (ARDS).^(4-6)^ Right ventricle failure (RVF) due to acute pulmonary hypertension may occur in up to 25% of severe ARDS patients.^(7)^ Nitric oxide and veno-venous extracorporeal membrane oxygenation (VV-ECMO) support are therapeutic options, but little has been discussed about further options in refractory cases.^(4-6)^

Here, we describe the use of balloon atrial septostomy^(8)^ - a procedure currently indicated in venoarterial ECMO (VA-ECMO) for left ventricle decompression - as a possible rescue therapy for RVF.

## CASE REPORT

A previously healthy 32-year-old female presented to the hospital with a 3-day history of shortness of breath and low fever. She had no history of smoking or environmental exposures, no recent travel or pets, and had two relatives at home reporting cough and fatigue. She suffered a car accident 5 weeks before the initiation of symptoms, with multiple vertebral fractures and bilateral fractures of the ischium and pubis. She underwent surgical correction of the fractures and was discharged home for rehabilitation after one week.

Three weeks after trauma, she was diagnosed with surgical site infection. Empirical treatment with teicoplanin and amikacin was initiated. She then underwent surgical debridement of the surgical site infection. Cultures of intraoperative soft tissue were positive for *Staphylococcus capitis* and *Staphylococcus lugdunensis*, which are methicillin-resistant and susceptible to trimethoprim-sulfamethoxazole. The antibiotic regimen was switched to a combination of trimethoprim-sulfamethoxazole and rifampicin, and she was discharged home after 12 days in the hospital.

After 5 days, she was admitted for tachypnea. dyspnea and respiratory distress, which were symptoms that were present for more than 72 hours. On admission, she had a respiratory rate of 30 breaths per minute and pulse oxygenation of 96% with a Venturi mask with a fraction of inspired oxygen (FiO_2_) of 32%. She had mild respiratory distress and reduced breath sounds in both lungs. Laboratory results were relevant for minor leukopenia (white blood cell count of 2.83 x 10^9^/L, 65% neutrophils, 29% lymphocytes, 7% monocytes, and 0% eosinophils). The complete metabolic panel was normal, and the HIV test was negative. Panel tests for influenza A and B, respiratory syncytial virus A and B, *Mycoplasma pneumoniae*, human metapneumovirus A and B, rhinovirus, enterovirus, parechovirus, coronavirus NL63, HKU, 229E and OC43, bocavirus, adenovirus, and parainfluenza 1, 2, 3 and 4 were all negative.

**Figure 1:**
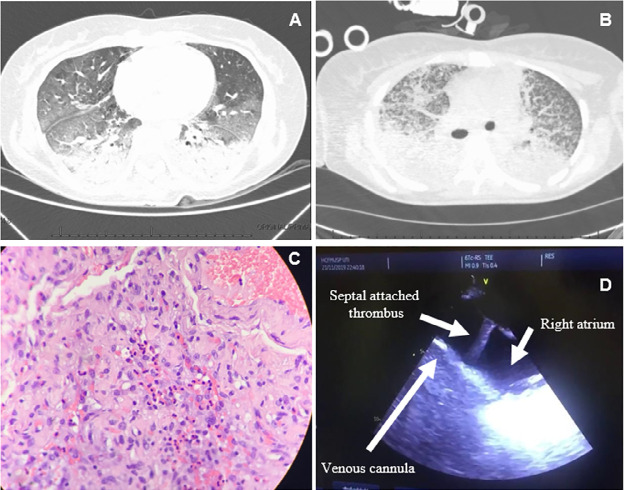
(A) Lung tomography at admission; (B) lung evolution after 14 days; (C) lung biopsy specimen with hematoxylin-eosin; (D) transesophageal echocardiography of the patient during a severe extracorporeal membrane oxygenation low blood flow episode.

Chest computed tomography (CT) revealed lung consolidations in the posterior and inferior areas bilaterally, with mixed ground glass and septal thickening and a few areas with mosaic pattern attenuation; the exam ruled out pulmonary embolism (Figure 1A). Despite supportive treatment, she developed progressive dyspnea and severe respiratory distress, and ten days after admission, she required mechanical ventilation (MV) and prone positioning for refractory hypoxemia. Extensive infective and autoimmune workups were negative. A new chest CT showed multiple micronodules that were not present before, in addition to worsening of the consolidations and interstitial infiltrate in both lungs (Figure 1B). She was started on methylprednisolone at a 1mg/kg dose on the hypothesis of drug-induced pneumonia. She continued to deteriorate, and after four days on MV, VV-ECMO support was initiated. An unfractionated heparin drip was placed to obtain an activated partial thromboplastin time (aPTT) of 2.5 - 3.5. She underwent open lung biopsy, which revealed interstitial thickening due to fibroblastic proliferation and chronic inflammatory infiltrate with eosinophils, areas with alveolar collapse, focal septal fibrosis, areas with multiple eosinophils, intraluminal edema and fibrin thrombi in smalland medium-caliber arteries, suggestive of acute eosinophilic pneumonia (Figure 1C). Routine and special histochemical stains were negative. There was no eosinophilia in the peripheral blood. A diagnostic hypothesis of eosinophilic pneumonia was made, and treatment with methylprednisolone 1g per day for 3 days, followed by 1mg/kg per day.

She was weaned from MV and was maintained on a high-flow nasal cannula at 40L O_2_/minute with FiO_2_ = 100% and ECMO support at a blood flow of 4.5L/minute, rotation of 3095rpm, sweep of 6.0, and FiO_2_ of 100% to maintain a pulse oxygen saturation of 90%. After six days on ECMO, she developed severe hypoxemia and was placed on mechanical ventilation again. The ECMO flow was low due to drainage insufficiency, with progressive negative pressures. Point-of-care ultrasonography showed normal biventricular function with a dilated inferior vena cava (IVC). A transesophageal echocardiogram revealed a misplaced venous cannula sucking the interatrial septum (Figure 1D). The venous cannula was repositioned, and the obstruction was resolved. However, a large thrombus attached to the interatrial septum was revealed. Anticoagulation was continued, with an aPTT = 3.5.

Her clinical status improved moderately at first, but she continued to need mechanical ventilation with high ECMO support. Two days later, eosinophilia was noted for the first time during the disease course, with 770 eosinophils in the peripheral blood.

**Figure 2:**
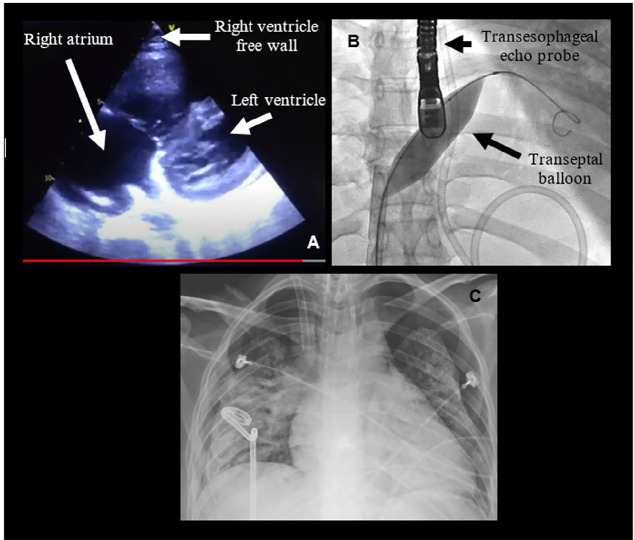
(A) Transthoracic echocardiography after the installation of severe pulmonary hypertension; (B) atrioseptostomy procedure; (C) thoracic X-ray depicting bilateral pneumothorax.

On the following days, she developed an acute cor-pulmonale, with echocardiography showing progressive right ventricular dilatation and dysfunction, with clinical deterioration (Figure 2A). At this point, her pulmonary systolic arterial pressure (PSAP) was estimated to be approximately 100mmHg. The patient was initiated on nitric oxide due to right ventricular failure and shock, with only a partial response in hemodynamics, and PSAP persisted above 100mmHg.

As a rescue maneuver for RVF, a balloon atrial septostomy was then performed through fluoroscopy and transesophageal echocardiogram guidance (Figure 2B), with an opening of a 12mm x 4mm communication between the atria to allow right-to-left blood flow. During the procedure, there was thrombus migration from the right atrium to the left ventricle outflow tract, with systemic embolization. Postprocedure transesophageal echocardiogram still showed significant distention of the right atrium and ventricle with the interventricular septum moving toward the left ventricle and severe tricuspid regurgitation, but PSAP decreased to 65mmHg, with a right atrial pressure (RAP) of 15mmHg. Immediately after the procedure, nitric oxide was no longer necessary, and all clinical signs of cor pulmonale resolved.

For the following 2 weeks, her clinical status remained stable. She continued to need mechanical ventilation and ECMO assistance to maintain a peripheral oxygen saturation of approximately 60%, but she was awake and had no signs of organ dysfunction or hemodynamic compromise. There was no evidence of improvement of lung function after 9 weeks of the presumed drug exposure and 6 weeks of the initiation of symptoms. No complications of systemic emboli were observed. She was not considered for pulmonary transplantation because of the local criteria at that time.

Six weeks after admission, the patient began to fluctuate in neurologic status, with periods of somnolence and mental confusion; her eosinophilia returned, reaching 2,410 eosinophils in peripheral blood. She presented a right spontaneous pneumothorax, with no compromise on oxygenation or hemodynamics. Despite thoracic drainage, the right lung did not expand. One day later, routine chest imaging revealed spontaneous pneumothorax in the left lung. Again, the left lung did not expand after thoracic drainage, suggesting a severe degree of alveolar damage and fibrosis that led to bilateral pneumothorax with no lung expansion (Figure 2C). At this point, the heparin infusion had to be stopped due to bleeding through the chest tube orifices. Her neurologic status then rapidly worsened, presumably due to central nervous system emboli, and she died 49 days after hospital admission.

## DISCUSSION

This is a case of balloon atrial septostomy for acute refractory cor-pulmonale in a patient on VV-ECMO due to eosinophilic pneumonia. The procedure is less invasive than converting to VA-ECMO, but complications may still occur. The most dangerous complication is systemic embolization to the central nervous system, since thrombus formed in the venous system may migrate to the left atrium, as occurred in our patient. The worsening of hypoxemia is expected in intracardiac shunts, and dealing with low oxygen saturations must be considered before the procedure.

Right ventricle failure is a common complication in patients with acute respiratory distress syndrome requiring ECMO, with significant associated mortality.^(9)^ However, its management is challenging and requires prompt intervention. The approach to refractory cor-pulmonale includes other short-term use ECMO configurations.

Balloon atrial septostomy was first described in 1966 by Dr. William Rashkind as a palliative treatment for transposition of the great vessels (TGV) in neonates,^(7)^ alleviating hypoxemia and RVF, and allowing survival until definitive surgery. In adults, Rashkind’s procedure is currently used for patients with pulmonary arterial hypertension (PAH) refractory to optimal medical therapy.^(10)^ Similar to its first description, the rationale is to decompress the right ventricle to ameliorate cor pulmonale symptoms. VV-ECMO support is described as a preemptive measure to avoid RVF in patients awaiting lung transplantation. The procedure is frequently used for patients on VV-ECMO complicated by left ventricle distension.

In this case, we used balloon atrial septostomy for RV decompression in the setting of refractory acute cor pulmonale. The beneficial effects on hemodynamics and clinical symptoms we observed in this case are consistent with those described in PAH patients.^(8)^ We conclude that for patients with RVF who are ineligible for other options of RV support, this may represent a therapeutic option while waiting for recovery, decision or bridge-to-transplant.
